# Rare paraneoplastic syndrome of prostatic cancer: limbic encephalitis: a case report

**DOI:** 10.1186/s13256-021-02975-3

**Published:** 2021-07-28

**Authors:** Omar Karray, Sven Tolner, Naïm Yarak, Maguy Cherfan, Mihaela Dana Cosma, Walid Sleiman, Philippe Niclot, Jean Louis Dubost, Patrick Coloby, Stéphane Bart

**Affiliations:** 1 Urology Department, René Dubos Hospital, 6, Avenue de l’Ile-de-France, 95300 Pontoise, France; 2 Neurology Department, René Dubos Hospital, Pontoise, France; 3 Pathology Department, René Dubos Hospital, Pontoise, France; 4 Intensive Care Unit, René Dubos Hospital, Pontoise, France

**Keywords:** Prostatic neoplasms, Limbic encephalitis, GABA receptors, Seizures

## Abstract

**Introduction:**

Limbic encephalitis is an autoimmune neurologic disorder, often of paraneoplastic origin, that seldom complicates prostatic tumors. The nonspecificity of symptoms makes the diagnosis sometimes difficult to establish. Prognosis is essentially determined by comorbidities and sensorineural and cognitive sequelae.

**Clinical case:**

A 66-year-old Caucasian patient known to have prostatic small-cell neuroendocrine adenocarcinoma under hormonal therapy developed complex partial epileptic seizures associated with rapidly aggravating severe memory impairment.

The tripod of autoimmune limbic encephalitis diagnosis was based on the clinical aspect of brain’s functional deterioration, electroencephalography aspect, and γ-aminobutyric acid type B anti-receptor antibody positivity. Clinical, diagnostic, and therapeutic management as well as evolutionary risks were further analyzed.

**Conclusion:**

Limbic encephalitis is an extremely rare presentation of neurologic paraneoplastic syndromes.

A better knowledge of this entity would help better manage diagnostic and therapeutic difficulties and reduce the risk of possible sequelae.

## Background

Limbic encephalitis is a rare autoimmune neurologic syndrome, frequently of paraneoplastic origin, caused by humoral and cellular response against intra- or extracellular specific neural antigens. Multiple antibodies responsible for limbic encephalitis were described and are mostly of paraneoplastic origin.

Limbic encephalitis is often associated with small-cell lung cancer [1], but also other cancers, particularly testicular and ovarian neoplasms.

It is usually revealed by acute or subacute temporal epileptic seizures, anterograde memory impairments, and neuropsychiatric disorders (depression, irritability, behavioral disorders, delusion, and hallucination).

Clinical manifestations, linked to limbic structures deterioration, may not be evident from the early onset of the disorder, making diagnosis very challenging.

Neurologic paraneoplastic syndromes, and to a lesser extent limbic encephalitis, scarcely occur in prostate cancer.

Some cases of limbic encephalitis linked to prostatic tumors were described in association with specific antibodies, such as anti-Hu anti-VGKC, anti-Ma-1, and anti-Ma-2 [2, 3].

Type B γ-aminobutyric acid receptor antibodies (GABA_B_) were recently described as responsible for the autoimmune encephalitis and, in particular, limbic encephalitis [2].

The ambiguity of the symptoms and the myriad of antibody panel, as well as the patient’s condition, which is often old aged with an advanced tumor pathology, highlight the diagnostic difficulties and the treatment response’s progression.

This case has been reported in line with the CARE guidelines [4].

## Case presentation

A 66-year-old Caucasian patient has been followed since 2004 for a Gleason score 8 (4 + 4) prostate adenocarcinoma, initially classified T3N0M0, treated by radiotherapy in association with luteinizing hormone-releasing hormone (LH-RH) analogs for a period of 3 years.

His family history was relevant for epilepsy in his two sisters.

Hormonal treatment was resumed in 2009 for biological recurrence, and he was then put under diethylstilbestrol in 2013.

In May 2017, he underwent prostate laser photovaporization for severe voiding disorders refractory to medical management. It should be noted that his prostate-specific antigen (PSA) back then was 1.53 ng/ml.

In September 2017, the patient presented generalized tonic–clonic seizures, followed by postictal confusion.

Initial neurological examination, standard laboratory tests, and brain imaging [brain scanner and magnetic resonance imaging (MRI)] did not show any abnormalities.

Treatment by levetiracetam was then introduced but stopped rapidly.

A few days later, he was hospitalized following the recurrence of the generalized epileptic crises. His wife reported memory and concentration problems that appeared after the first epileptic episode, along with an escalating depressive syndrome.

During his hospitalization course, the patient suffered from confusion, phasic disorder with slurred speech, and disorders of simple and complex order comprehension in addition to partial seizures, manifested by loss of contact, chewing, sniffing, clonism, and right gaze deviation.

**Fig. 1:**
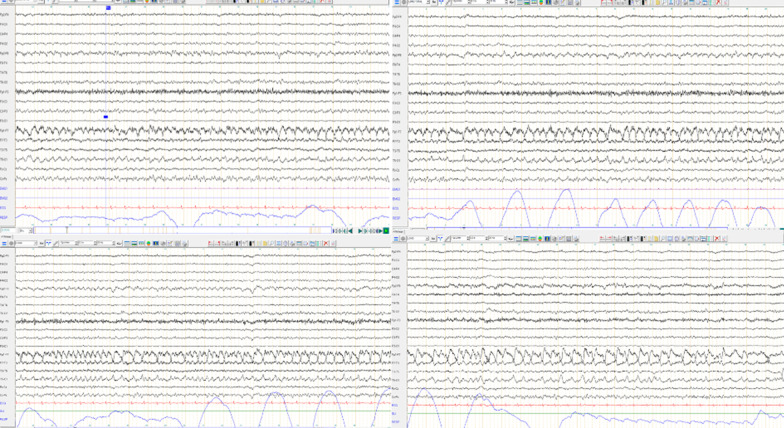
Example of left temporal seizure with rhythmic discharges of left temporal slow waves and slow spikes

Epileptic activity with focal onset in left frontotemporal region, secondarily generalized, was documented by the electroencephalogram (EEG) shown in Fig. 1.

Intravenous administration of clonazepam and levetiracetam allowed clinical improvement, with, however, persistence of left temporal interictal slow peaks on control EEG.

Cerebrospinal fluid (CSF) analysis demonstrated normal cytology, glycorrhachia, and protein rate. Bacterial culture and herpes virus polymerase chain reaction (PCR) in the CSF were both negative.

Furthermore, supernumerary bands of immunoglobulin were found on the CSF analysis with the absence of Hu, Ri, and Yo neural antibodies. It is noteworthy to mention that GABA_B_ anti-receptor antibodies were positive in the CSF, thus evoking the diagnosis of limbic encephalitis with GABA_B_ receptor antibodies.

The patient was then hospitalized in the intensive care unit for 2 weeks secondary to the aggravation of the epileptic seizures, where he was intubated with thiopental sedation after failure of phenytoin and phenobarbital effect.

Methylprednisolone treatment was then started with three boluses of 1 g, followed by treatment with polyvalent immunoglobulin 2 g/kg over 5 days when autoimmune limbic encephalitis diagnosis was confirmed. Antiepileptic treatment had to be escalated gradually by levetiracetam, clobazam, lacosamide, and carbamazepine.

The course of his hospitalization was then marked with successful extubation after 7 days along with a few episodes of fluctuating agitation. The control EEG patterns improved gradually with striking evidence of resolved generalized status epilepticus and persistent foci of seizures from right and left temporal starting point. However, some left temporal slow puff of waves persisted without seizure before his discharge from the intensive care unit.

**Fig. 2:**
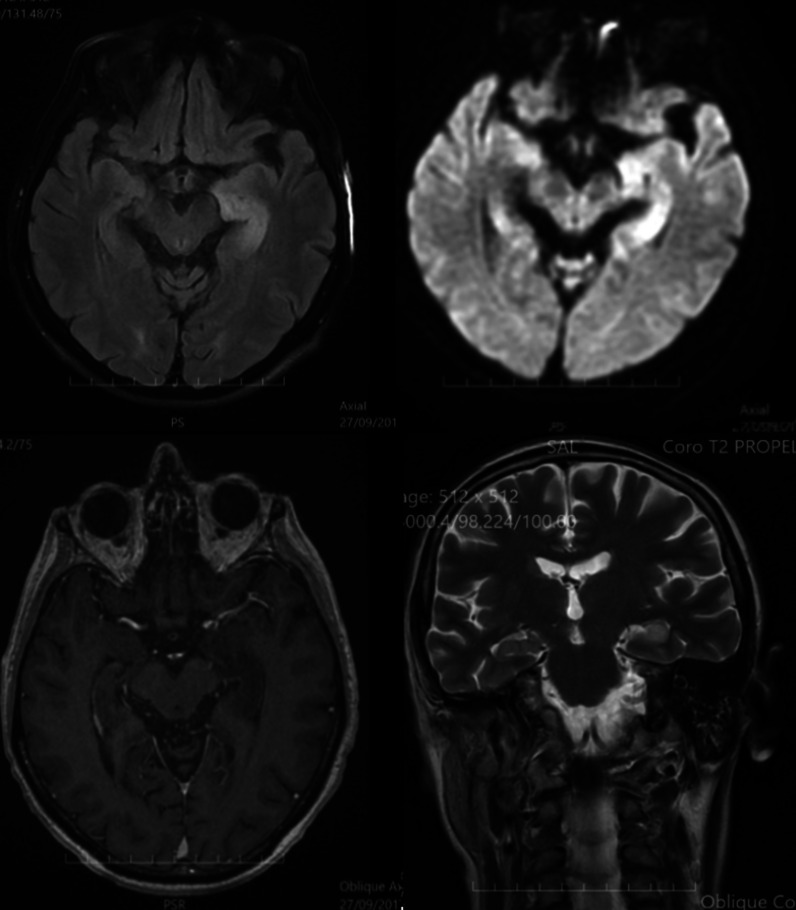
MRI: left hippocampal and parahippocampal hypersignal, without contrast enhancement

Brain MRI was performed 3 weeks after the first epileptic seizures showing left hippocampal and parahippocampal hypersignal in T2 and fluid-attenuated inversion recovery (FLAIR) sequences, with swelling appearance, without contrast enhancement, compatible with the initial diagnosis of limbic encephalitis (Fig. 2).

Choline positron emission tomography (PET) scan showed, besides the prostate and iliac ganglionic areas uptake, left temporal brain hypermetabolism corresponding to the electric epileptic focus and the hypersignal observed on MRI. Considering that GABA_B_ anti-receptor antibodies limbic encephalitis is frequently associated with small-cell lung tumors, bronchoscopy with bronchial aspiration were performed, showing no evidence of malignancy.

Back in the neurology department, the patient was calm and better oriented without any sensorimotor deficits or phasic disorders. However, mild ataxia and especially massive anterograde memory disorders persisted. Neuropsychological assessment revealed verbal and visual anterograde memory disorders as well as psychomotor fatigue and concentration difficulties.

Hospitalization was complicated with left lower limb deep vein thrombosis treated by curative anticoagulation.

Immunotherapy was changed to immunosuppressive treatment.

The patient received one course of cyclophosphamide of 1 g and two courses of rituximab of 375 mg/m^2^.

No prolongation of cyclophosphamide treatment was done owing to persistent neutropenia and lymphopenia. Rituximab was not renewed 6 months later as treatment with chemotherapy was established.

Control brain MRI showed stable left hippocampal and parahippocampal T2 and FLAIR hypersignal, without contrast enhancement.

The patient then remained neurologically stable for 2 years, with visual anterograde and immediate memory improvement. Nevertheless, verbal anterograde memory was deficient with autobiographical lacunar amnesia regarding the 2 years that preceded the disorder.

Epileptic seizures receded with normalization of the subsequent control EEG patterns and tapering of antiepileptic treatment.

Concerning the prostate cancer follow-up, diethylstilbestrol was stopped and was replaced by degarelix before tumor progression on imaging. It should be recalled that the PSA level at the beginning of the neurological events was 0.16 ng/ml, then increased to 1.1 ng/ml, and then dropped back to 0.68 ng/ml 6 months after degarelix was introduced.

Following voiding urinary symptoms complaints, several investigations were conducted, including abdominopelvic ultrasound showing a bilateral ureteral and pyelocalyceal dilatation with a postvoid residual of urine of 300 cc with laboratory data indicating the start of kidney injury with creatinine level of 140–150 µmol/l serum.

Flexible cystoscopy revealed an obstructive prostate. Consequently, prostate endoscopic bipolar resection was performed.

**Fig. 3:**
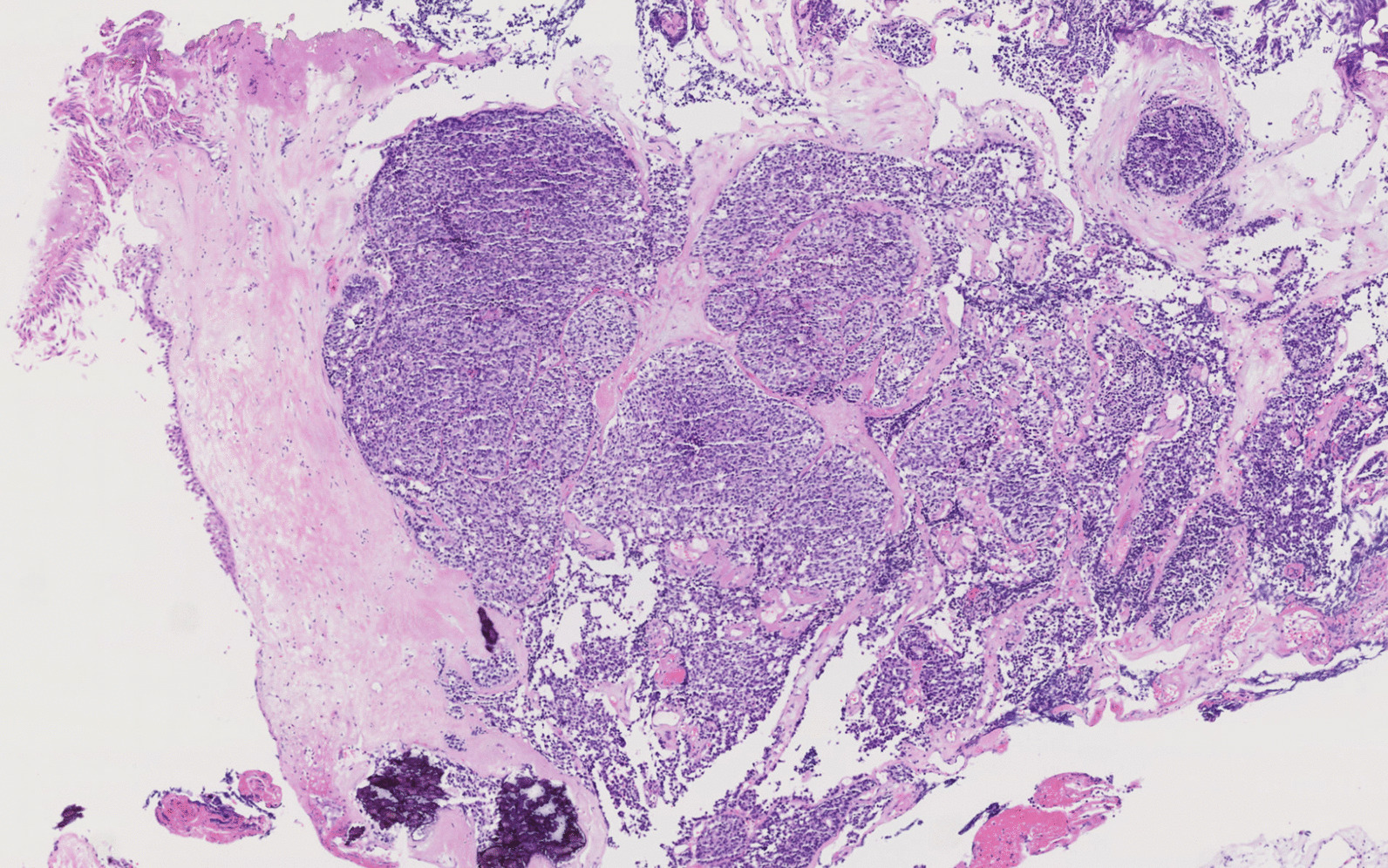
Microscopic histological examination; hematoxylin–eosin coloration: poorly differentiated carcinomatous proliferation, with significant necrotic eosinophilic change

Histological examination found a poorly differentiated carcinomatous proliferation, with significant eosinophilic necrosis. Carcinomatous cells were observed in ranges and sheets of average size, presenting hyperchromatic nuclei with irregular outlines and quite abundant cytoplasm (Fig. 3).

**Fig. 4:**
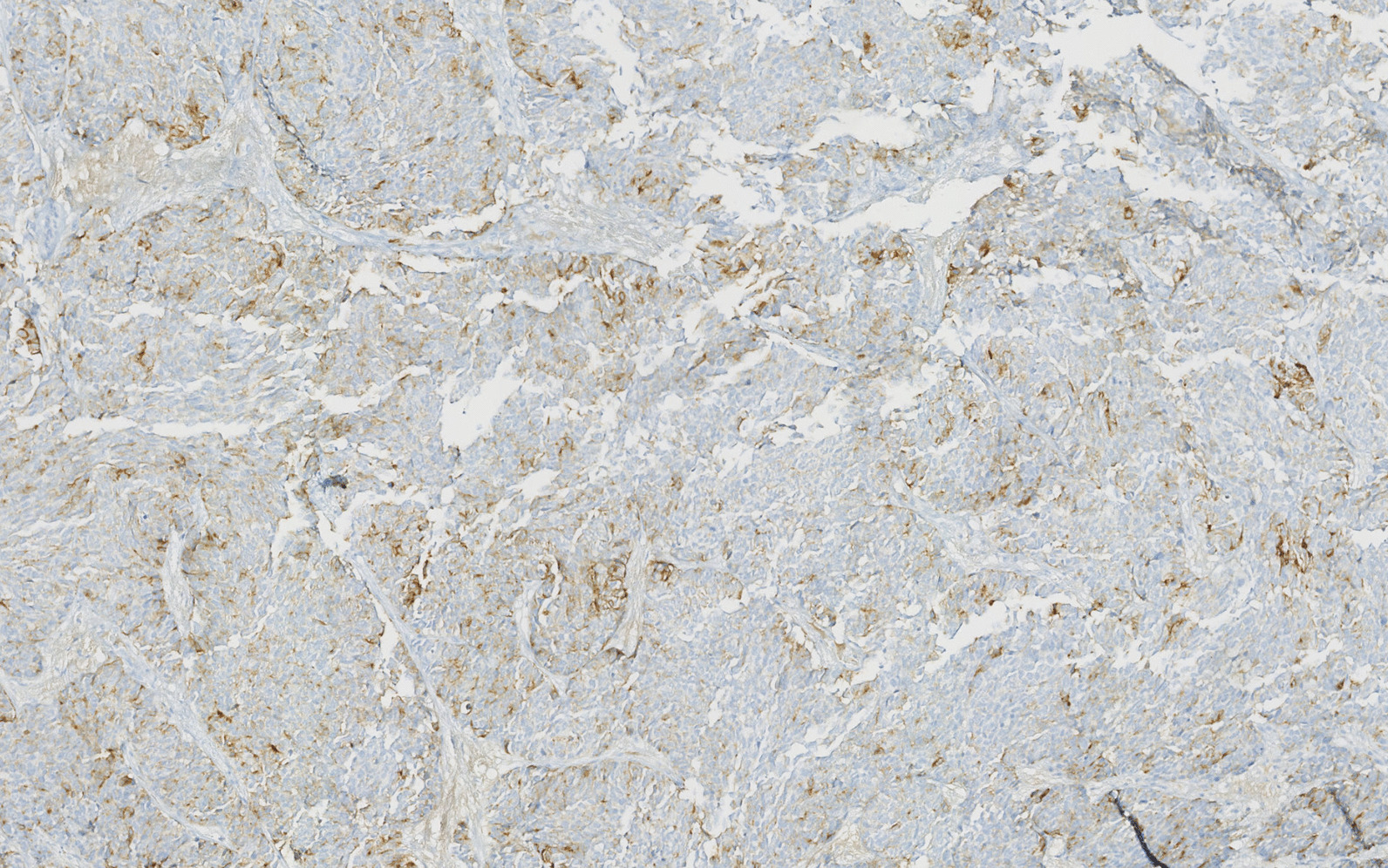
Immunohistochemical findings: positivity for neuroendocrine markers (chromogranin A)

**Fig. 5:**
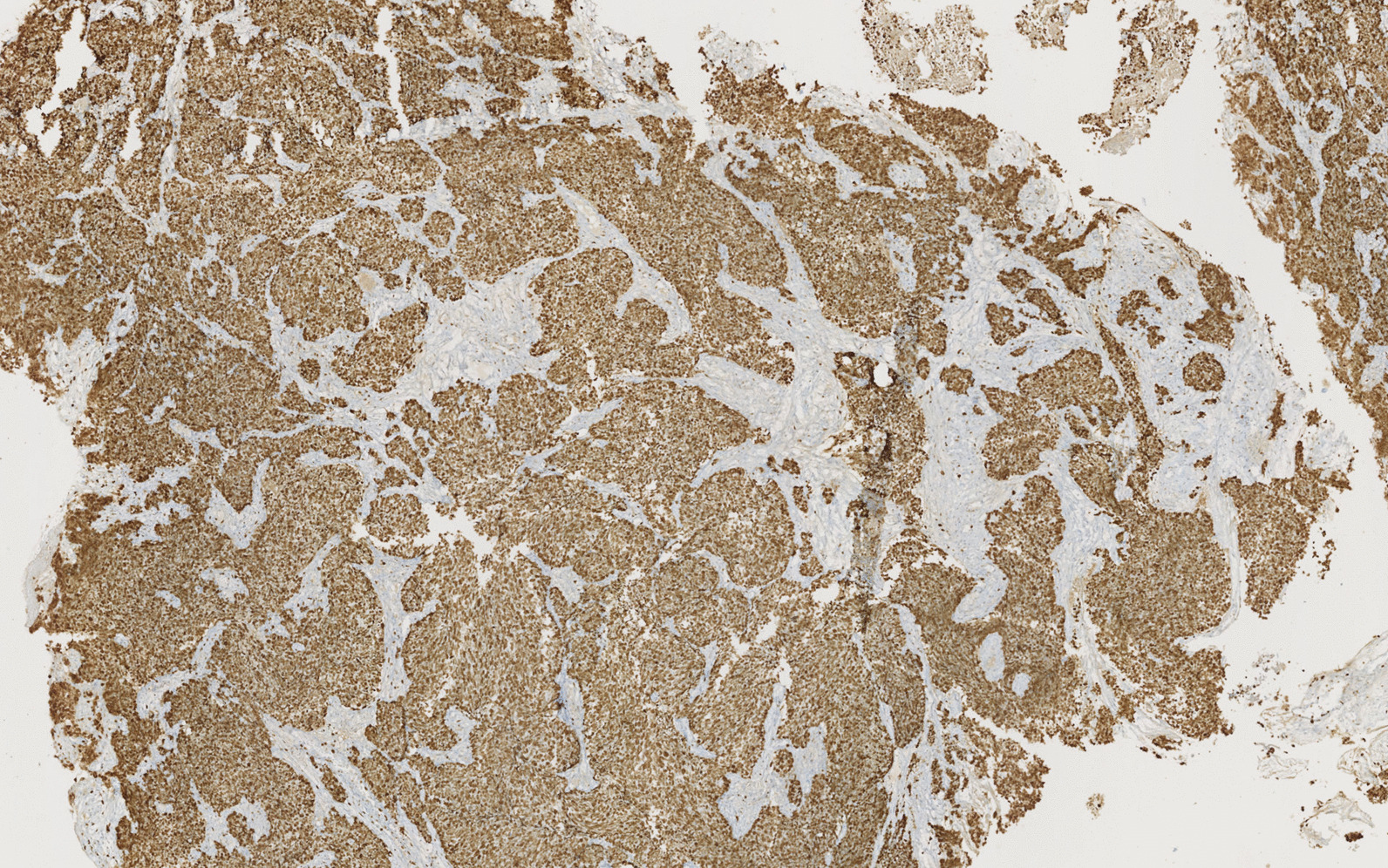
Immunohistochemical findings: High Ki67 proliferation index, marking 100% of tumor cells

Immunohistochemical examination showed positivity for neuroendocrine markers (CD56, synaptophysin, and chromogranin A), and absence of expression of CK7 and PSA (Fig. 4). Ki67 proliferation index was high, marking 100% of tumor cells (Fig. 5).

A multidisciplinary discussion was held, and decision was made to start chemotherapy of which he initially received six courses of cisplatin with etoposide after discontinuation of immunosuppressive treatment. PSA level after the last course was 0.21 ng/ml.

Follow-up PET scan showed the presence of subdiaphragmatic ganglia; accordingly, docetaxel as a second-line treatment was started with an unfavorable response after six courses evidenced by bilateral palpable indurated inguinal adenopathies and hepatic metastatic nodules on choline PET scan.

Hence, a third-line chemotherapy by doxorubicin associated with cyclophosphamide was started with progression of hepatic nodules on follow-up PET scan after three cycles.

As such, cabazitaxel as a last chemotherapy was commenced with initial favorable response after three courses. Subsequently, PET scan findings deteriorated, showing isotopic, ganglionic, bone, hepatic, and cerebral progression.

Considering the aggressive disease profile, it was decided to stop any further treatment and to confine the patient to palliative care.

## Discussion

Neurologic paraneoplastic syndromes are rarely associated with prostatic tumors.

Clinical aspects are variable. Beside limbic encephalitis, observations of cerebellar degenerations, inappropriate antidiuretic hormone secretion syndrome, or peripheral neuropathy were reported in a few case series [5].

Forms related to GABA antineuronal antibodies represent 5% of autoimmune encephalitis.

The development of immunological tests for serodiagnosis and antibody typing allowed the classification of the previously considered idiopathic or viral encephalitis, as autoimmune encephalitis, with well-defined immunological profiles [6].

Paraneoplastic encephalitis poses diagnostic and etiological problems. Clinically, symptom onset is often acute or subacute. The most frequently revealing sign is seizure.

Disorientation, delirium, aphasia, and memory disorders may be observed with variable degrees of severity.

Diffuse cerebral damage, cerebellar ataxia, and encephalomyelitis can worsen initial presentation in about 40% of cases [7].

Paraneoplastic encephalitis reveals primitive tumor in about 60% of the cases, all sites combined. The most frequently reported tumors are small-cell lung tumors [1].

Differential diagnosis is sometimes difficult to establish, given clinical resemblance with brain metastases and infectious encephalitis [8].

Paraclinical diagnosis is based on three essential elements: brain MRI, electroencephalogram patterns, and antineural antibody assays.

Brain MRI is more sensitive than scanning, as FLAIR sequences ensure higher detection of lesions. It concerns mainly temporal lobe hypersignal in T2 and FLAIR sequences [9]. If MRI is normal, PET scan can be useful. Temporal hypermetabolism can be explained by convulsions and acute inflammation [8].

Electrical modifications in temporal region are observed in half of the cases. EEG is particularly useful during follow-up [8].

CSF is abnormal in 80% of cases, showing pleocytosis, hyperproteinorachia, and possibly intrathecal immunoglobulin synthesis [8]. The rate of blood or cerebrospinal fluid antibodies is not correlated with clinical and evolutive profile [2].

The treatment should be targeted against both the encephalitis and the primitive tumor. The initial management consists essentially of antiepileptic treatment, parenteral corticotherapy, immunotherapy, and eventually plasmapheresis.

In the rare reported cases complicating prostate cancer, evolution was favorable in one case only with clinical and radiological stabilization following total prostatectomy for a localized cancer [10].

The first description of limbic encephalitis as a paraneoplastic syndrome complicating a primitive prostatic small-cell carcinoma was published by Stern in 1999 [11]. Symptoms at initial presentation included fever, lower urinary tract symptoms, and truncal ataxia in a 76-year-old patient. Evolution was rapidly unfavorable. Histological diagnosis was confirmed after autopsy.

Regarding GABA receptors, they were first described recently by Lancaster *et al.* in 2010 [9]. Alberti reports almost constant aberrant production of GABA in invasive neuroendocrine tumors [12]. The type of antibodies associated with neurological disorders determines in part the treatment efficiency. The forms with neuronal antibodies directed against intracellular antigens are less likely to evolve favorably. Antibodies acting on surface antigens, such as GABA receptor, have better prognosis under immunotherapy [13].

Lin *et al*. reported 28 cases of autoimmune encephalitis by GABA_B_ receptor antibodies [14].

Prognosis of paraneoplastic forms is more unfavorable. Mortality predictive factors are advanced age, paraneoplastic character, occurrence of septic complications, and deep vein thrombosis. Major causes of mortality in intensive care are tumor progression, severe pneumonia, epileptic seizure, and septic shock.

Early diagnosis and treatment of primitive tumor improve clinical course and prognosis.

The case we report is, to our knowledge, the first to be described with antineural antibodies against GABA_B_ receptor associated with prostate neuroendocrine tumor. The initial management of encephalitis required intensive care.

Corticotherapy treatment was relayed by immunoglobulin course first, then immunotherapy by rituximab and immunosuppressive treatment by cyclophosphamide.

Besides the initial neurological disorders, we particularly note the occurrence of deep venous thrombosis of the lower limb as an element of utmost gravity.

Despite the absence of epileptic seizure recurrence, neurocognitive sequelae and memory disorders persisted, particularly anterograde and autobiographical.

On the carcinological level and given the metabolic progression, diethylstilbestrol was replaced by degarelix. The nonelevation of PSA could be explained by the presence of neuroendocrine component on prostate resection specimen.

In fact, prostate neuroendocrine tumors, well known for their aggressiveness, are generally hormone-resistant. Tumor progression following the initiation of different chemotherapy lines, particularly seen on metabolic imaging, justified the decision of palliative care.

## Conclusion

Paraneoplastic encephalitis remains exceptionally underreported in prostate cancer. Diagnostic difficulty is related to the nonspecificity of initial symptoms, and the etiological investigation of the primitive tumor. Comorbidities, tumor evolution, and specific complications in intensive care affect prognosis. A better knowledge of physiopathological mechanisms with precise immunological typing permits adjustment of therapeutic strategies and improves evolution. The oncological management should be associated with initial measures. The best profiles are associated with curative management, when plausible.

## Data Availability

Authors ensure ready reproducibility and free availability of materials described in the manuscript to any scientist wishing to use them, without breach of confidentiality. Authors make materials described in the manuscript available for testing by reviewers in a way that preserves the reviewers’ anonymity.
